# Relationship between frontal knee position and the degree of thoracic kyphosis and lumbar lordosis among 10-12-year-old children with normal body weight

**DOI:** 10.1371/journal.pone.0236150

**Published:** 2020-07-29

**Authors:** Agnieszka Jankowicz-Szymańska, Michał Fałatowicz, Eliza Smoła, Renata Błyszczuk, Katarzyna Wódka

**Affiliations:** Faculty of Health Sciences, University of Applied Sciences in Tarnow, Tarnow, Poland; Instituto Federal Goiano, BRAZIL

## Abstract

**Introduction:**

Incorrect positioning of the body in space increases the tension of the myofascial tissue and overloads the skeleton. It is important to look for factors that affect the deterioration of body posture that could be eliminated. Understanding the interrelationship between the positioning of individual body segments should be the key knowledge for those involved in the prevention and correction of faulty body posture. The study aimed to determine the relationship between the degree of physiological curvatures of the spine and the incidence of incorrect knee position.

**Materials and methods:**

The study involved 685 children aged 10–12. Body height, weight and BMI were measured and calculated. The degree of thoracic kyphosis and lumbar lordosis was assessed using the Zebris Pointer ultrasound system. Valgus and varus knees were diagnosed in an upright position based on the intermalleolar distance with knees together, and intercondylar distance with the feet placed together. The statistical analysis uses descriptive statistics, the Mann-Whitney U test (comparison of girls and boys), the Kruskal-Wallis test, the Tukey's post hoc test (comparison of variables in participants with correct, varus and valgus knees) and Spearman's rank correlation coefficient (the relationship between the position of the spine and knees).

**Results:**

The examined girls were heavier than the boys and had higher BMI. Spine deformities and incorrect knee position are common among 10-12-year-old children. The girls and boys differed significantly in the spine shape in the sagittal plane and the intermalleolar distance. Round lumbar lordosis is more characteristic for girls, and for boys, round thoracic kyphosis. For both genders, valgus knees occur more often than varus knees and coexist with decreased thoracic kyphosis. The rounder the thoracic kyphosis, the greater distance between the knees and the smaller distance between ankles.

**Conclusions:**

The frontal knee position significantly correlated with the depth of thoracic kyphosis.

## Introduction

Correct body posture, i.e. the correct positioning of individual body segments, is required for the optimal efficiency of motor activities with minimal load on stabilizing structures [1]. Incorrect positioning of the body in the space increases the tension of the myofascial tissue and overloads the skeleton, which leads to functional disturbances, premature development of degenerative lesions and pain, but also to reduction of cardiovascular fitness, displacement of internal organs and reduction of quality of life [2–4]. Unfortunately, faulty body posture and the associated musculoskeletal dysfunctions are currently one of the biggest health problems in every age group. They affect about 50% of children and adolescents [5] and at least the same percentage of adults [6]. Systematic monitoring of body posture (implemented for children) should also be continued after a period of posture stabilization [7]. Factors that affect the deterioration of body posture, especially those that can be modulated, should also be determined. Such factors are, for example, excessive body weight and a sedentary lifestyle. Their adverse impact on body posture quality have been proven many times [8–11]. The position of individual body segments relative to each other is also important. The relationship between the position of the sacrum and the degree of physiological spine curvatures is known [12]. The relationship between the loss of lumbar lordosis and knee flexion as a compensatory mechanism for imbalance in sagittal plane has also been documented [13, 14]. Tsuji et al [15] even describe the correlation between sacral inclination and patellofemoral joint pain as the knee-spine syndrome. There is no doubt that sagittal body imbalance (which is affected by knee alignment in the same way as it is by spine alignment) correlates with reduced quality of life and increased pain [16]. However, there is a lack of research on the relationship between the position of the spine in the sagittal plane and valgus and varus knee positions, although the current knowledge about the myofascial network indicates the possibility of such links. Myofascial restriction can weaken muscles and change the alignment of the skeletal elements [17]. For example, the gluteus maximus, which is connected to the thoracolumbar fascia and the fascia of the thigh, can affect knee position [18, 19]. Understanding the relationship between the positioning of individual body segments is the key for those involved in the prevention and correction of faulty body posture.

We aimed to assess the relationship between the degree of physiological curvatures of the spine and the incidence of incorrect knee position among 10-12-year-old girls and boys with normal body weight. Any confirmation of such a correlation would help to understand the mechanism of faulty posture and develop an effective therapy plan using existing compensatory mechanisms. The age of the participants (10–12 years) was chosen because of the physiological process of knee maturation. Physiological varus knees are observed in children up to 2 years of age, whereas children between 3 and 6 years of age have physiological valgus knees. At the age of 10, the knees should be set to the neutral position [20].

## Materials and methods

### Participants

The study was conducted from mid-September to the end of November 2018 in accordance with the principles set out in the Declaration of Helsinki prepared by the World Medical Association and the consent of the bioethics committee at the District Medical Association was obtained (approval number 2/0177).

The group under study consisted of children between 10 and 12 years old. Participation in the study was voluntary. Written consent of parents or legal guardians was required. Nine hundred and one children expressed their willingness to participate in the study. To limit the effect of excessive body weight on the results of the study, overweight and obese children were excluded from the analysis (206 cases). The status of body weight was determined on the basis of the BMI index. The thresholds determining excessive body weight were taken from the studies of Cole et al. [21]. Overweight or obesity in 10-year-old boys was diagnosed when the BMI was 19.84 or 24.00, respectively, in 11-year-olds for BMI of 20.55 or 25.10, and in 12-year-olds for BMI of 21.22 or 26.02. Overweight or obesity in 10-year-old girls was diagnosed when the BMI was 19.86 or 24.11, in11-year-olds for BMI of 20.74 or 25.42, 12-year-old for BMI of 21.68 or 26.67.

The study also excluded children (10 cases) with disabilities confirmed by a specialist doctor, those treated for chronic diseases, and those who had a serious injury to the musculoskeletal system (sprain or fracture). Information on the children's health was obtained from their parents. All participants were characterised by good overall health. Ultimately, the group included in the study consisted of 685 children.

### Study tools and procedures

An anthropometer (ZPH Alumet No 010208, Warsaw, Poland) was used to measure body height. The measurement was carried out with an accuracy of 0.1 cm, from the basis point to the vertex point in a standing position, with the feet placed together and eyes facing forward.

A Tanita weighing scale (body composition analyzer bf-350; Tanita Corporation of America, Inc., Arlington Heights, Illinois) was used to measure body weight. The measurements were made with an accuracy of 0.1 kg.

**Fig 1:**
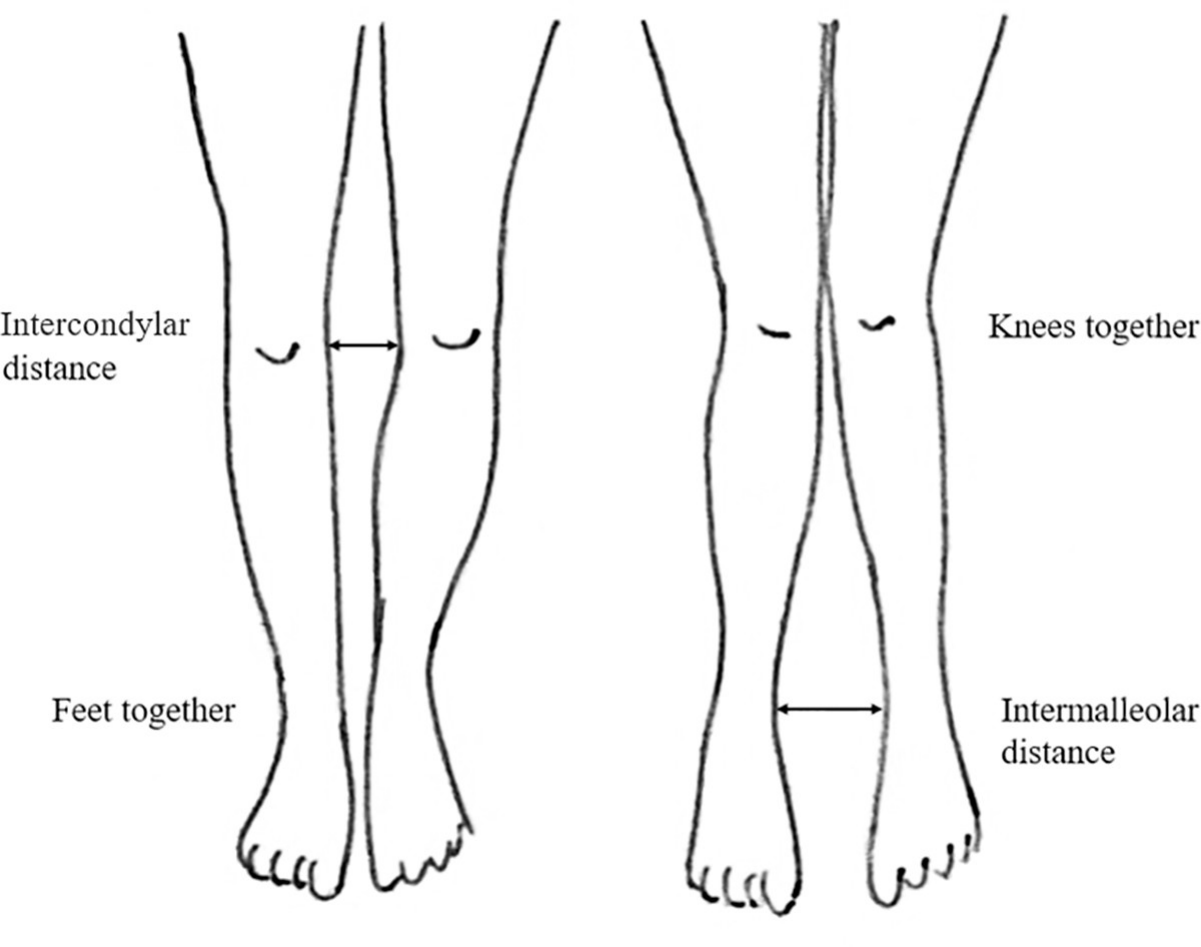
Frontal knees position measurement (intermalleolar distance of 2.5 cm or more were considered as valgus knees and intercondylar distance of 2.5 cm or more as varus knees).

The position of the lower limbs was examined in a barefoot standing position with the knees fully extended. Valgus knees were determined by measuring the intermalleolar distance (IMD) with knees together and varus knees were determined by measuring the intercondylar distance (ICD) with the feet placed together. A spreading caliper (ZPH Alumet No 030208, Warsaw, Poland) was used for the measurements. The measurement accuracy was 0.1 cm (Fig 1). The method was chosen because of its simplicity, reliability and it did not need to expose the children in the study to X-ray radiation [22–24]. The children in the study were at an age when neither valgus or varus knees are no longer a physiological norm. For this reason, IMD and ICD values of 2.5 cm or more were considered abnormal [25,26].

**Fig 2:**
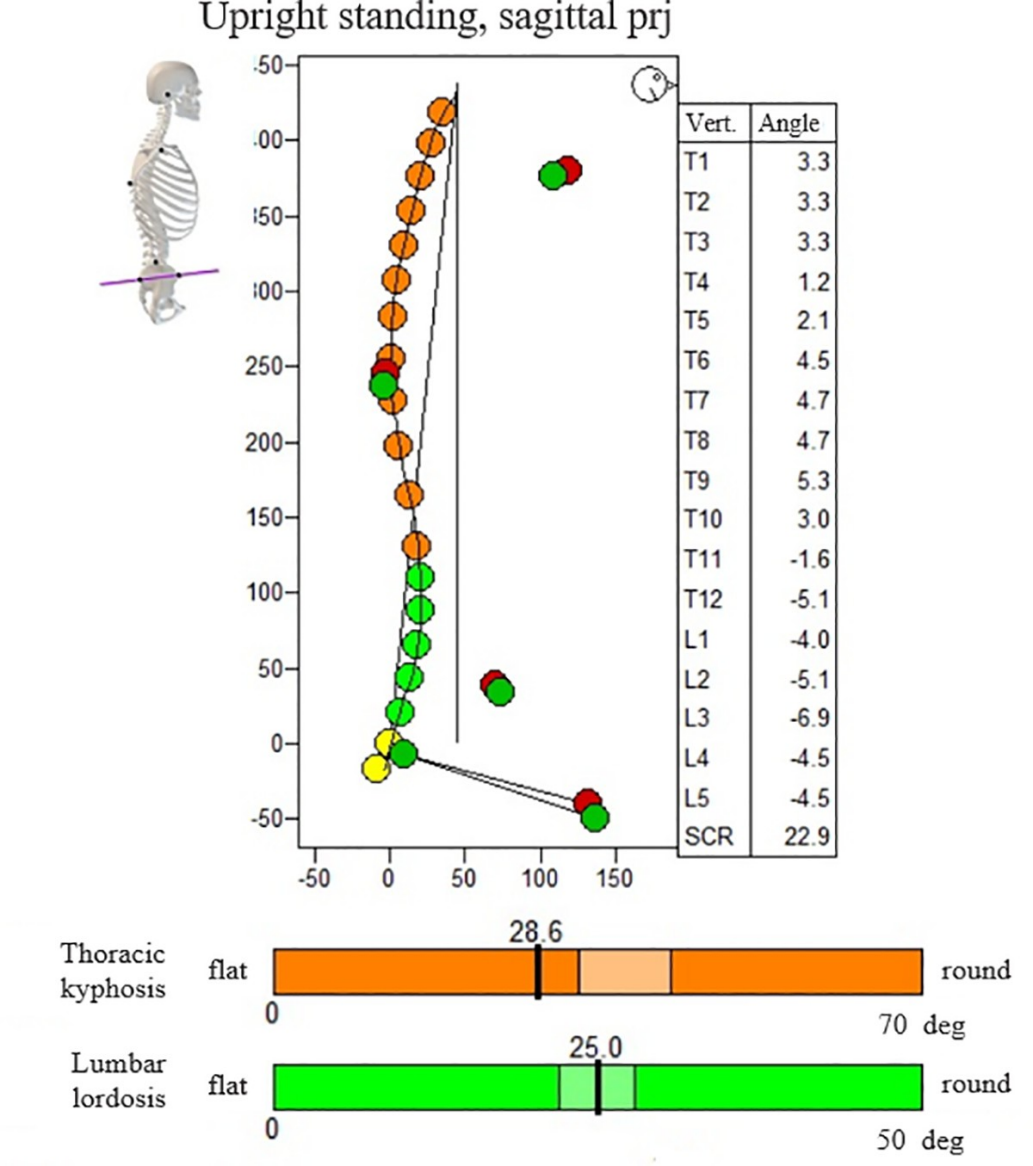
Zebris pointer report—spinal curvatures measurement (the thresholds determining flat and round thoracic kyphosis and lumbar lordosis).

The size of thoracic kyphosis and lumbar lordosis was examined using the Zebris Pointer ultrasonic system (Zebris Medical GmbH Company, Germany), which consists of a basic unit (it measures the position of the markers and transmits the data to the computer), an ultrasonic pointer (used for marking anatomical points on the study subject's body), a reference marker (it eliminates fluctuations of the participant's position occurring during measurement), a tripod and WinSpine software. The test was performed in a habitual standing position. The therapist, using an ultrasonic pointer, marked selected anatomical points on the participant's body in a strictly defined order: the acromions of the shoulder blades, the posterior superior and anterior superior iliac spines, the tops of the wings of the ilia, the inferior angles of the scapula and the thoracolumbar junction. Then the spinolaminar line from C7 to the sacrum was marked three times (Fig 2). These data were used to determine the angle of thoracic kyphosis (the total angle was formed from the sum of the angles of all the thoracic vertebrae) and lumbar lordosis (the total angle was formed from the sum of the angles of all the lumbar vertebrae). Using the standards developed by the Physical Medicine Clinic of the University of Munich, thoracic kyphosis and lumbar lordosis were classified as flat, normal or round. It was assumed that the normal value of thoracic kyphosis is 33^o^-43^o^ in men and 21^o^-32^o^ in women, while the normal value of lumbar lordosis is 22^o^-28^o^ in men and 28^o^-34^o^ in women [27]. The reliability and usefulness of the Zebris Pointer system in assessing spine curvature has been confirmed by researchers [28–30].

### Statistical analyses

For the data analysis, carried out using the Statistica v13 program, descriptive statistics were used for participants' demographic and clinical data. The Shapiro-Wilk test was used to examine the normality of the variable distribution, while the F test was used to assess the homogeneity of the variance. The Mann-Whitney U test was used to assess the significance of the differences between the two groups, and the Kruskal-Wallis test and Tukey's post hoc test were used for more than two groups. The relationship between the variables was examined using Spearman's rank correlation coefficient. The correlation was considered weak for R <0.4 moderate for R in the range of 0.4–0.69 and strong for R = or > 0.7 [31, 32]. A significance level α = 0.05 was adopted.

Sample size calculation showed that, with the mean values we observed, to show a significant difference in the depth of thoracic kyphosis between participants with valgus and correctly positioned knees, a count of 158 would suffice. To show a significant difference in the depth of thoracic kyphosis between participants with varus knees and correctly positioned or valgus knees, the minimum sample size should be 198. To show significant difference in the depth of lumbar lordosis between groups divided according to knee position, the estimated minimum sample size should be 675 participants.

## Results

Among the participants were 351 girls (51.24%) and 334 boys (48.76%) and in the particular age groups 233 were 10-year-olds (117 girls and 116 boys), 239 were 11-year-olds (121 girls and 118 boys) and 213 were 12-year-olds (113 girls and 100 boys). The number of participants divided by frontal knees alignment was: valgus knees n = 216, normal knees position n = 411, varus knees n = 58. The boys and girls in the study did not differ significantly in terms of age, body height and intercondylar distance. The girls were 1.45 kg heavier than the boys and their mean BMI was 0.44 kg/m^2^ higher. There were also significant differences in the intermalleolar distance, which was 0.51 cm greater among the girls, and the depth of both physiological curves. Thoracic kyphosis was greater among the boys by 3.09^o^, and lumbar lordosis greater among the girls by 1.73^o^ (Table 1).

**Table 1:** Comparison of studied variables among girls and boys.

Variable	Gender	Mean	Median	Min-Max	St Dev	p
Age [years]	Girls	10.99	11.00	10.00–12.00	0.81	0.579
	Boys	10.95	11.00	10.00–12.00	0.81	
Body height [cm]	Girls	147.08	147.00	128.40–167.50	8.76	0.251
	Boys	146.34	145.70	122.40–170.50	8.20	
Body weight [kg]	Girls	37.78	37.30	21.00–57.60	7.63	0.013*
	Boys	36.33	35.55	21.10–57.60	6.50	
BMI [kg/m^2^]	Girls	17.29	17.41	12.56–21.43	2.09	0.004*
	Boys	16.85	16.84	11.85–21.20	1.85	
Intercondylar distance [cm]	Girls	0.42	0.00	0.00–6.00	1.00	0.168
	Boys	0.56	0.00	0.00–6.00	1.16	
Intermalleolar distance [cm]	Girls	1.89	1.00	0.00–9.00	2.16	0.002*
	Boys	1.38	0.50	0.00–9.50	1.84	
Thoracic kyphosis [˚]	Girls	37.45	38.10	0.00–67.10	12.01	0.001*
	Boys	40.54	40.80	0.00–67.30	12.16	
Lumbar lordosis [˚]	Girls	26.63	27.90	0.00–50.00	11.43	0.008*
	Boys	24.90	25.00	0.00–50.00	10.98	

* statistically significant difference

Girls had the same incidence of flat, normal and round kyphosis. Boys had a round kyphosis almost twice as often as a flat one. Valgus knees were diagnosed more often than varus knees for both genders. It was also observed that valgus knees for both genders were more often present in participants with flat rather than round thoracic kyphosis. This tendency was more pronounced among boys (Table 2).

**Table 2:** The incidence of incorrect knee alignment among study participants with flat, normal and round thoracic kyphosis.

Gender	Thoracic kyphosis	Valgus knee n (%)	Normal knee n (%)	Varus knee n (%)	Row totals n (%)
Girls	flat	44 (37.93%)	60 (51.72%)	12 (10.34%)	116 (33.05%)
	normal	43 (35.83%)	68 (56.67%)	9 (7.50%)	120 (34.19%)
	round	38 (33.04%)	72 (62.61%)	5 (4.35%)	115 (32.76%)
Boys	flat	28 (35.90%)	46 (58.97%)	4 (5.13%)	78 (23.35%)
	normal	29 (25.00%)	73 (62.93%)	14 (12.07%)	116 (34.73%)
	round	34 (24.29%)	92 (65.71%)	14 (10.00%)	140 (41.92%)
Column totals		216	411	58	685

Almost half of the girls were diagnosed with round lumbar lordosis. The incidence of round and flat lumbar lordosis was similar among boys. The incidence of varus knees does not appear to be related to the shape of the lumbar spine (Table 3).

**Table 3:** The incidence of incorrect knee alignment among study participants with flat, normal and round lumbar lordosis.

Gender	Lumbar lordosis	Valgus knee n (%)	Normal knee n (%)	Varus knee n (%)	Row totals n (%)
Girls	flat	33 (32.67%)	60 (59.41%)	8 (7.92%)	101 (28.77%)
	normal	20 (25.97%)	50 (64.94%)	7 (9.09%)	77 (21.94%)
	round	72 (41.62%)	90 (52.02%)	11 (6.36%)	173 (49.29%)
Boys	flat	28 (23.73%)	80 (67.80%)	10 (8.47%)	118 (35.33%)
	normal	24 (26.37%)	58 (63.74%)	9 (9.89%)	91 (27.25%)
	round	39 (31.20%)	73 (58.40%)	13 (10.40%)	125 (37.42%)

There was a significant relationship between the depth of thoracic kyphosis and intercondylar and intermalleolar distances among the entire population under the study and in the boys’ group. It was observed that the rounder the thoracic kyphosis, the greater distance between the knees and the smaller distance between the ankles. The value of the correlation coefficient shows that the relationship, although significant due to the level of p, was weak (Table 4).

**Table 4:** Relationships between the depth of thoracic kyphosis and lumbar lordosis and the intercondylar distance and intermalleolar distance in the studied girls and boys.

Correlated variables	Gender	R	p
Thoracic kyphosis & ICD	Girls	0.003	0.942
	Boys	0.143	0.008*
	All	0.086	0.024*
Thoracic kyphosis & IMD	Girls	-0.095	0.074
	Boys	-0.126	0.021*
	All	-0.125	0.001*
Lumbar lordosis & ICD	Girls	-0.049	0.358
	Boys	-0.001	0.979
	All	-0.030	0.428
Lumbar lordosis & IMD	Girls	0.030	0.574
	Boys	0.030	0.589
	All	0.041	0.278

* statistically significant correlation, ICD–intercondylar distance, IMD–intermalleolar distance, R—correlation coefficient

In the group under study, a significant smaller angle of thoracic kyphosis was observed among children with valgus knees compared to children with the correct knee position. The degree of lumbar lordosis did not differ in the groups under study for valgus, varus or correctly positioned knees (Table 5).

**Table 5:** The depth of thoracic kyphosis and lumbar lordosis among participants with valgus, normal or varus knees.

Variable	Knee position	Mean	Median	Min-Max	St Dev	p
Thoracic kyphosis [˚]	Valgus n = 216	37.20	38.75	0.70–65.70	12.15	VL vs N p = 0.02* VL vs VR p = 0.30 VR vs N p = 0.98
	Normal n = 411	39.80	40.20	0.00–67.30	12.20	
	Varus n = 58	39.53	36.11	17.50–62.80	11.50	
Lumbar lordosis [˚]	Valgus n = 216	26.64	28.20	0.00–50.00	11.45	VL vs N p = 0.38 VL vs VR p = 0.72 VR vs N p = 0.99
	Normal n = 411	25.39	25.50	0.00–50.00	11.30	
	Varus n = 58	25.37	26.20	0.00–50.00	9.87	

* statistically significant difference, VL–valgus knee, VR–varus knee, N–normal knee position

## Discussion

Two-thirds of the 10-12-year-olds under study were diagnosed with incorrect spinal alignment in the sagittal plane. Round lumbar lordosis was more characteristic for girls, and round thoracic kyphosis for boys. Incorrect frontal knee position affected about 40% of respondents, with valgus knees more frequent than varus knees for both genders (valgus vs. varus knee was 3 vs. 1 in boys and 5 vs. 1 in girls). The most important observation resulting from our study is the significant correlation between flat thoracic kyphosis and valgus knee position as well as between varus knees and increased thoracic kyphosis.

In the study of Maciałczyk-Paprocka et al. [8], in a group of 888 boys and girls aged 7–12 with normal body weight, round thoracic kyphosis, round lumbar lordosis and flat back were diagnosed much less frequently than in our study (7.1%, 6.6% and 1.8%, respectively). However, this study was carried out using another method (visual assessment of body posture), which may explain the differences. The tendency for round thoracic kyphosis among boys and round lumbar lordosis among girls was also observed by Chromik et al. [33]. According to Mellin and Pous, who examined 294 children aged 8–16 [34], girls not only have significantly flatter thoracic kyphosis compared to boys, but also a smaller range of movement of forward and lateral flexion. However, no effect of gender on the degree of thoracic kyphosis of 345 10-11-year-olds was observed, using the rasterstereography system, by other researchers [35]. At the same time, they found that the angle of lumbar lordosis was only slightly higher among girls compared to boys. Erkan et al. [36] also believe that gender does not differentiate the depth of thoracic kyphosis. It should be noted, however, that study concerned adults. The Zebris ultrasound system was used by Walicka Cupryś et al to assess spinal curvature. They examined 109 7-year-old children and noted a similar mean value of lumbar lordosis (about 25^o^) and a slightly higher mean value of thoracic kyphosis (about 45^o^) as in our study. The findings of that study did not compare thoracic kyphosis and lumbar lordosis in girls and boys [37].

In a properly developing child, the varus knee position is observed in the newborn and infant period until the child starts to walk on its own. Then, the knees spontaneously position themselves valgusly in preschool aged children and also spontaneously and gradually correct themselves to a neutral position after 7 years of age [38]. If the valgus or varus knee position remains for a longer period than the physiological norm indicates, or is severe, then biomechanical compensatory mechanisms appear. For example, varus knees increase the internal rotation of the knee and the external rotation of the hip. This changes the position of the foot and gait [39, 40]. Both valgus and varus knees significantly overload the knee joint, change the patella position in the patellofemoral joint and increase the risk of ligament damage [41].

In the presented study, varus knees were diagnosed among 7.41% of girls and 9.58% of boys. Studies show that at the age of 3–7 this deformity occurs less frequently (up to about 2%) [42] and among 13-18-year-olds in about 9% of the population, slightly more often among boys [43]. Valgus knees in the present study were diagnosed in one in three girls and one in four boys. This is more than in the studies of Maciałczyk-Paprocka [43], who observed valgus knees among 16% of 7-12-year-olds. However, more rigorous standards were applied in our study.

No recent research were found about relationship between an incorrect frontal knee position and incorrect spine alignment among school children. In our study, we observed that 10-12-year-old children with valgus knees have significantly flatter thoracic kyphosis and slightly rounder lumbar lordosis compared to their peers with normal and varus knee positions. This observation may be justified in the theory of anatomy trains, which states that changes in the position of one section of the body by overloading the myofascial structures change the load on the segments above and below [44]. This theory is confirmed by the research of Khamis and Yizhar [45], in which it was shown that immediately after standing on a specially prepared wedges platform, which forces pronation of the feet, the thigh is positioned in an internal rotation and the pelvis in an anterior tilt.

Confirmation of our observations would be of great practical importance. It would broaden knowledge about biomechanical and functional compensatory mechanisms and would mean that care for proper frontal knee positioning is an important element of preventing spinal deformities in the sagittal plane. However, the results presented should be approached with due caution. This is the first study of this type and should be carefully compared with current knowledge about myofascial chains. There are several concepts about myofascial chains, of which the best known are the theories of Tom Myers [44], Carla Stecco [45] and the concept of G.D.S. [46]. In each of these, connections of the tissues from the trunk area with muscles and the fascia of the lower limb were noted. This means that these structures interact, but none of them clearly explains the impact of lower limb positioning on the position of the spine.

### Study limitations

The study limitations are the research methods used. Certainly, the gold standard method would be to use X-rays. We decided not to use this method for ethical reasons. According to the researchers, there was no need to expose children to the adverse effects of radiation. Another recommended method for assessing the position of the spine and knees is 3-dimensional (or 2-dimensional) movement analysis. However, this method is expensive, time consuming and requires a lot of space. Conducting a research using this method would be very difficult with such a large group of children [47]. Radiological, photographic and clinical methods are often used in research, but it is difficult to compare their results. Therefore, conclusions about the similarities or differences obtained should be cautious.

Another limitation was the smaller number of children with varus knees. This was due to the lower occurrence of this misalignment in the study population.

## Conclusions

Imbalance of the sagittal spine curvatures and incorrect frontal knee positions are common among 10-12-year-old children. Girls tend to have round lumbar lordosis and boys tend to have round thoracic kyphosis. Valgus knees are more common than varus ones for both genders. There are significant relationships between the depth of physiological curvatures of the spine and the position of the knees. Valgus knees more often occur with flat thoracic kyphosis.

### Clinical implication

The results of our research show that in clinical practice taking care to ensure the correct positioning of the knee can be an important element in correcting spine deformities in the sagittal plane. Future research that would confirm these relationships is necessary.

## Supporting information

S1 Data (STA)
